# Atherosclerotic risk is associated with cerebral perfusion – A cross-sectional study using arterial spin labeling MRI

**DOI:** 10.1016/j.nicl.2022.103142

**Published:** 2022-08-04

**Authors:** Melanie Hafdi, Henk JMM Mutsaerts, Jan Petr, Edo Richard, Jan Willem van Dalen

**Affiliations:** a Department of Neurology, Amsterdam University Medical Center, Amsterdam, The Netherlands; b Department of Radiology and Nuclear Medicine, Amsterdam University Medical Center, Amsterdam, The Netherlands; c Helmholtz-Zentrum Dresden-Rossendorf, Institute of Radiopharmaceutical Cancer Research, Dresden, Germany; d Department of Public and Occupational Health, Amsterdam University Medical Center, Amsterdam, The Netherlands; e Department of Neurology, Donders Institute for Brain, Cognition and Behaviour, Radboud University Medical Center, Nijmegen, The Netherlands

**Keywords:** Arterial spin labeling MRI, Atherosclerosis, Cerebral perfusion, Spatial coefficient of variation, White matter hyperintensity volume, ASCVD, AtheroSclerotic CardioVascular Disease, ASL, Arterial Spin Labeling, ATT, Arterial Transit Time, BMI, Body Mass Index, CBF, Cerebral Blood Flow, CVD, CardioVascular Disease, CI, Confidence Interval, *EPI*, Echo-Planar Imaging, FEAST, Flow Encoding Arterial Spin Tagging, FLAIR, Fluid-Attenuated Inversion Recovery, FOV, Field Of View, GM, Gray Matter, HDL, High Density Lipoprotein, LDL, Low Density Lipoprotein, LST, Lesion Segmentation Tool, MNI, Montreal Neurological Institute, MRI, Magnetic Resonance Imaging, PCASL, Pseudo-Continuous Arterial Spin Labeling, PLD, Post-Labeling Delay, PreDIVA, Prevention of Dementia by Intensive Vascular Care

## Abstract

**Background:**

Arterial spin labeling (ASL) magnetic resonance imaging (MRI) may be a promising technique to evaluate the presence of cerebral atherosclerosis. We tested whether the new and easily calculated ASL MRI parameter for vascular and tissue signal distribution - ‘spatial coefficient of variation’ (ASL-sCoV) - is a better radiological marker for atherosclerotic risk than the more conventional markers of white matter hyperintensity (WMH) volume and cerebral blood flow (ASL-CBF).

**Methods:**

Participants of the preDIVA trial (n = 195), aged 72–80 years with systolic hypertension (>140 mmHg) underwent two MRI scans two to three years apart. WMH volume was derived from 3D FLAIR-MRI; gray matter ASL-CBF and ASL-sCoV from ASL-MRI. Atherosclerotic risk was operationalized as 10-year cardiovascular risk by the Systematic COronary Risk Evaluation Older Persons (SCORE O.P) and calculated at baseline and follow-up. Data were analyzed using linear regression.

**Results:**

ASL-CBF was associated with atherosclerotic risk scores at baseline (standardized-beta = -0.26, 95 %CI = -0.40 to −0.13, p < 0.001) but not at follow-up (standardized-beta = -0.14, 95 %CI = -0.33 to 0.04, p = 0.12). ASL-sCoV was associated with atherosclerotic risk scores at both time points (baseline standardized-beta = 0.23, 95 %CI = 0.10 to 0.36, p < 0.0001, follow-up standardized beta = 0.20, 95 %CI = 0.03 to 0.36, p = 0.02). WMH volume was not associated with atherosclerotic risk scores at either time-point. There were no longitudinal associations between changes in MRI parameters and baseline atherosclerotic risk scores.

**Conclusions:**

Our findings suggest that ASL-sCoV correlates better with atherosclerotic risk than the more conventional markers ASL-CBF and WMH volume. Our data reaffirm that non-invasive imaging with MRI is highly informative and could provide additional information about cerebrovascular damage.

## Highlights

• Non-invasive MRI can provide additional information on atherosclerotic risk.
• ASL-sCoV correlates better with atherosclerotic risk than more conventional markers.
• Longitudinal change in MRI markers is not associated with atherosclerotic risk.

## 1 Introduction

Chronic atherosclerosis can cause cerebrovascular damage through extra- and intracranial stenosis and cerebral arteriolar occlusive disease (Pantoni, 2010, Kalback et al., 2004). Increased vascular resistance in the cerebral arterioles caused by atherosclerosis may contribute to cerebral hypoperfusion and cerebral small vessel disease, with reduced oxygen supply to brain tissue due to disrupted cerebral blood flow, and ultimately leading to neurodegeneration and cognitive decline (Kivipelto et al., 2001, Yaffe et al., 2014, Peers et al., 2009).

Non-invasive magnetic resonance imaging (MRI) may be a promising technique to evaluate the presence of subclinical cerebral atherosclerosis by quantifying well-established presentations of small vessel disease, such as white matter hyperintensity (WMH) volume, or measuring cerebral blood flow (CBF). CBF may be more sensitive to earlier stages of atherosclerosis, as WMHs may develop after the long-term presence of atherosclerosis, while reductions in CBF might be apparent more immediately (Aljondi et al., 2020). CBF can be measured as a single quantitative image using arterial spin labeling (ASL-CBF). It takes a short time, called the arterial transit time (ATT), for the labeled blood to reach the imaged voxel. After a post-labeling delay (PLD), which is ideally longer than the ATT, the ASL signal in brain tissue is imaged and quantified as a ASL-CBF map (Alsop, 2015). To accurately assess ASL-CBF, the PLD has to be long enough for the blood to spread from the large vessels to the tissue perfusing microvasculature, yet short enough to make sure that the ASL label is still accurately measurable, as ASL label decays over time. However, the ATT differs between participants and brain regions, especially in the presence of atherosclerosis, and the ASL labeled blood can still be in larger vessels rather than in tissue at the time of imaging. This causes heterogeneity in ASL images and less accurate estimation of ASL-CBF (Mutsaerts et al., 2017). Moreover, ASL-CBF has a high intra- and interindividual physiological variability, making it less suitable as parameter for high vascular resistance in individuals (Zhang et al., 2002, Henriksen et al., 2018, Liu et al., 2016). Recently, the innovative ASL-derived parameter ‘spatial coefficient of variation’ (ASL-sCoV) has been introduced that can estimate ATT indirectly from a single PLD ASL image based on signal heterogeneity (Mutsaerts et al., 2017). Recent literature suggests that the distribution of ASL signal – as measured throughout ATT or sCoV – could even be more informative about cerebral vasculature than its tissue perfusion component ASL-CBF (Mutsaerts et al., 2020).

In this study among community-dwelling older adults, we aim to examine the correlation of different MR imaging parameters (i.e. WMH volume, gray matter (GM) ASL-CBF and ASL-sCoV) with atherosclerosis, operationalized by the individual’s atherosclerotic risk score. Additionally, we will also investigate whether baseline atherosclerotic risk correlates with longitudinal changes on MRI. We hypothesize that ASL-sCoV correlates better as a radiological marker with atherosclerotic risk scores than the more conventional imaging parameters WMH volume and ASL-CBF and that high baseline atherosclerotic risk is associated with a decrease in ASL-CBF and increase in ASL-sCoV over time.

## 2 Material and methods

### 2.1 Study design and participants

**Fig. 1:**
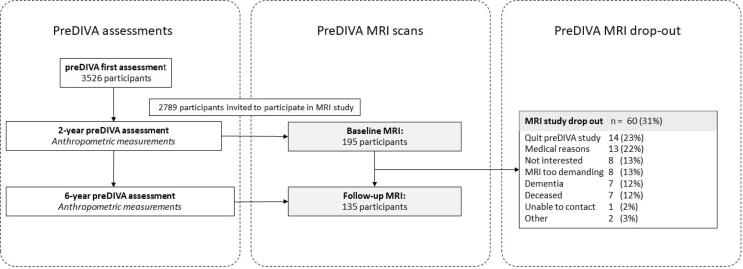
Flowchart of the study.

We performed a posthoc analysis on the MRI substudy of the Prevention of Dementia by Intensive Vascular Care (PreDIVA-M) Trial (Fig. 1). The preDIVA trial and the preDIVA-M substudy have been described in detail previously (Moll van Charante et al., 2016, Dalen, 2017). In brief, the preDIVA trial was a multicenter, cluster-randomized controlled trial that studied the efficacy of a nurse-led intervention program aimed at vascular risk factor modification among community-dwelling older adults. The main outcome was all-cause dementia after 6 years of follow-up.

A consecutive subset of participants from the preDIVA cohort with systolic hypertension (>140 mmHg) and without dementia at baseline was invited to participate in the preDIVA-M substudy. In total, 195 participants were included in preDIVA-M, equally distributed across the intervention and control group of the preDIVA trial. We considered the trial population as a single cohort irrespective of treatment allocation because the trial intervention did not have any effect on the parameters of interest in the current study (i.e. WMH volume, ASL-CBF, ASL-sCoV) (Moll van Charante et al., 2016, Dalen, 2017). A second MRI scan was performed two to three years after the first MRI scan.

The preDIVA trial was approved by the medical ethics committee of the Academic Medical Center, Amsterdam, the Netherlands. All participants provided written informed consent before their baseline visit. MRI substudy participants gave additional written informed consent before MRI.

### 2.2 MRI acquisition

All imaging was acquired using identical scanning parameters on 3 T Intera (baseline) and Ingenia (follow-up) MRI scanners from Philips (Philips Healthcare, Best, the Netherlands), equipped with a SENSE eight-channel head coil, using identical scanning protocols. A 1x1x1 mm^3^ 3D T1-weighted sequence and a 1x1x1 mm^3^ 3D fluid-attenuated inversion recovery (FLAIR) sequence were performed. Two consecutive background-suppressed gradient-echo *EPI* pseudo-continuous ASL (PCASL) sequences were acquired (matrix, 64x64; repetition time (TR)/echo time (TE), 4000/17 ms; flip angle, 90°; field of view (FOV), 240x240 mm; 17 axial sections; no gap; 7-mm section thickness; resolution 3.75x3.75x7mm^3^; SENSE factor, 2.5; post-labeling delay of the first slice = 1525 ms, slice readout time = 34.9 ms, post-labeling delay of the last slice = 1525 + 16*34.9 = 2080 ms; labeling duration, 1650 ms) with and without vascular flow-crushing gradients in three directions (ASL-CBF crushed: b-value = 0.6 s/mm^2^, equivalent to velocity-encoding 50 mm/s; ASL-CBF non-crushed: b-value = 0 s/mm^2^).

### 2.3 Assessment of brain volumes and ASL-CBF

Studied MRI parameters included WMH volume, GM volume, total brain volume (TBV), ASL-CBF in GM, and ASL-sCoV. WMH volume was calculated from FLAIR images using an automatic segmentation algorithm especially trained and validated for this dataset (Steenwijk et al., 2013). TBV was calculated by adding white matter (WM) and GM volumes from Statistical Parametric Mapping (SPM) 12 segmentation of T1-weighted images (Ashburner, 2012). Image processing for the structural and ASL images was performed with ExploreASL (Mutsaerts et al., 2020). Structural processing employed lesion segmentation tool (LST)-based WMH lesion-filling of the T1w (Schmidt et al., 2012) and CAT12 (T1w segmentation and spatial normalization) (Ashburner, 2012, Franke et al., 2010). GM and WM masks were defined as pGM > 0.7 and pWM > 0.7, where the WM mask was eroded threefold to isolate the deep WM and avoid contamination of WM with GM signal (Mutsaerts et al., 2014). ASL image processing included motion correction, motion outlier detection, and rigid-body registration of ASL-CBF to the pGM map (Mutsaerts et al., 2018). ASL-CBF was quantified from ASL images using a single compartment model for a single-PLD according to the ASL consensus review (Alsop, 2015). We used a mean deriver M0-value obtained previously from the same sequence to calibrate the scans. All quantified CBF images were transformed into the Montreal Neurological Institute (MNI) space. Population analysis was performed on the data transformed to MNI space with 1.5x1.5x1.5 mm^3^.

ASL-sCoV was defined as the standard deviation of ASL-CBF divided by the mean ASL-CBF in the entire GM mask (Mutsaerts et al., 2017). T1w and FLAIR images were visually assessed for motion artifacts, after which we discarded T1w and FLAIR images of one scan. ASL images were visually assessed for motion and labeling artifacts, after which we discarded ASL images of nine scans.

### 2.4 Assessment of atherosclerotic risk

We operationalized atherosclerotic risk with the Systematic COronary Risk Evaluation Older Persons (SCORE O.P.) in our main analysis and assessed the robustness of our findings in secondary analyses using the Framingham and the AtheroSclerotic CardioVascular Disease (ASCVD) risk scores (Cooney et al., 2016, D'Agostino, 2008, Lloyd-Jones et al., 2017). Using atherosclerotic risk scores as a proxy for actual atherosclerosis is a novel approach, although several studies have demonstrated that simple cardiovascular risk scores are significantly associated with the presence of MRI-detected subclinical cerebrovascular disease (Song et al., 2020, Anand et al., 2019). The SCORE O.P. predicts 10-year cardiovascular disease (CVD) mortality risk and was calculated using age, total cholesterol, high-density lipoprotein (HDL) cholesterol, systolic blood pressure (SBP), smoking status, and history of diabetes for each participant at baseline and follow-up (Cooney et al., 2016). The Framingham Heart Study CVD risk score predicts 10-year risk for CVD events and was calculated using age, history of diabetes, smoking status, treated and untreated SBP, total cholesterol, and HDL cholesterol at baseline and follow-up (D'Agostino , 2008). The ASCVD predicts the 10-year risk for atherosclerotic CVD and was longitudinally calculated using age, sex, race, total cholesterol, HDL cholesterol, SBP, blood pressure-lowering medication use, diabetes status, and smoking status, using risk factor data measured at the time of the scan and the closest preceding study visit, approximately 2 years earlier (Lloyd-Jones et al., 2017). For all three risk equations, a higher score indicates a greater risk of future cardiovascular events.

### 2.5 Statistical analyses

We assessed the cross-sectional association of WMH volume, ASL-CBF in GM (crushed and non-crushed), and ASL-sCoV (crushed and non-crushed) with atherosclerotic risk scores at baseline and follow-up separately using linear regression models. Additionally, we investigated the associations of atherosclerotic risk scores at baseline with both absolute longitudinal change in MRI parameters and MRI parameters at follow-up. Effect sizes were standardized to facilitate comparison between radiological parameters and were reported as standardized-beta with 95 % confidence interval (CI). In line with previous studies, we performed a logarithmic transformation on WHM volume and ASL-sCoV, to correct the skewed distribution of these variables (Mutsaerts et al., 2017, van Dalen et al., 2016, Silbert et al., 2012). MRI parameters in model 1 were unadjusted except for WMH and GM volume, which were both adjusted for total brain volume to account for more room for WMH and GM in larger brain volumes. In model 2, analyses were additionally adjusted for history of CVD and/or stroke and in model 3, analyses were additionally adjusted for covariates that are associated with atherosclerosis but were not included in the SCORE O.P., i.e. ethnicity, body mass index (BMI), low-density lipoprotein (LDL), and diastolic blood pressure. We did not adjust for parameters included in the SCORE O.P. to prevent over attribution of effect to these parameters due to high multicollinearity. However, as the SCORE O.P. was greatly influenced by age, its age-dependent associations with ASL-CBF may become excessively driven by increasing age itself rather than by the other vascular risk factors. Therefore, we performed a sensitivity analysis excluding age from the SCORE O.P. computation to investigate if our findings would remain consistent.

Other sensitivity analyses included: 1) exclusion of participants with a history of CVD and stroke, as atherosclerotic risk scores are formally not suitable for these participants; 2) exclusion of participants on baseline that did not attend the follow-up scan, to investigate whether the different composition of the population attending baseline and follow-up compared to attending baseline only influenced results for the main analyses; 3) imputation of missing SCORE O.P. values using predictive mean matching within the R mice package v3.13.0 (van Buuren and Groothuis-Oudshoorn, 2011); 4) exclusion of outliers in WMH and GM volume, ASL-CBF in GM, and ASL-sCoV, operationalized according to Tukey's definition as less than Q1 – (1.5 times the interquartile range (IQR)) or more than Q3 + (1.5 times the IQR) as these values could have a disproportional large influence on the linear regression results (Tukey, 1977). To check the robustness of our findings operationalizing atherosclerotic risk using the SCORE O.P., we additionally compared WMH and GM volume, ASL-CBF in GM, and ASL-sCoV with atherosclerotic risk defined by the Framingham and ASCVD risk score. All analyses were carried out in R statistical software version 3.6.3 (R Core Team. R, 2019). We used the Strengthening The Reporting of OBservational Studies in Epidemiology (STROBE) cross-sectional checklist when writing our report (von Elm et al., 2008).

## 3 Results

Of the 195 participants who underwent MRI at baseline, 135 (69 %) had a follow-up scan, on average 34 months later. The mean age of the population at the baseline scan was 77 ± 2.5 years, 47 % were male. Vascular and imaging parameter characteristics of the cohort are presented in Table 1. 26 % of the participants had a history of CVD or stroke at baseline. The mean SCORE O.P. was 14 % risk (±8) of 10-year CVD at baseline and 34 % risk (±15) in the 135 participants at follow-up.

**Table 1:** Participant and scan characteristics.

	**Baseline**	**Follow-up**
Scans, n	195	135
**Demographics**		
Age, years	77.1 (2.5)	80.1 (2.6)
Sex, Male, n (%)	92 (47.2)	64 (47.4)
**Vascular risk factors**		
Systolic blood pressure, mmHg	161.4 (15.5)	156.4 (18.4)
Diastolic blood pressure, mmHg	84.7 (10.0)	79.6 (11.0)
Cholesterol, mmol/l	5.5 (1.2)	5.2 (1.1)
HDL, mmol/l	1.5 (0.4)	1.6 (0.5)
LDL, mmol/l	3.4 (1.0)	2.8 (1.0)
DM, n (%)	18 (9.2)	18 (14.0)
BMI, kg/m^2^	26.7 (3.7)	25.5 (4.2)
Smoker, n (%)	16 (8.2)	6 (4.4)
**Medication use**		
Antihypertensives, n (%)	79 (40.5)	75 (55.6)
Antithrombotics, n (%)	47 (24.1)	43 (31.9)
Antiplatelets, n (%)	44 (22.6)	35 (25.9)
**Medical history**		
History of CVD, n (%)	39 (20.7)	26 (19.8)
History of stroke, n (%)	13 (6.8)	10 (7.5)
History of CVD or stroke, n (%)	49 (26.1)	34 (26.2)
**Imaging parameters**		
TBV in mL	1046.1 (105.1)	1029.4 (104.9)
WMH volume in mL, median (IQR)	6.4 (3.6–11.2)	8.0 (4.4–13.6)
ASL-CBF GM in mL/100 g/min, non-crushed	79.4 (18.3)	80.3 (24.4)
ASL-CBF GM in mL/100 g/min, crushed	67.3 (21.9)	64.6 (24.5)
ASL spatial CoV, non-crushed, median (IQR)	0.48 (0.41–0.57)	0.46 (0.42–0.55)
ASL spatial CoV, crushed, median (IQR)	0.46 (0.40–0.58)	0.44 (0.40–0.54)
**10-year cardiovascular risk scores**		
SCORE O.P.	14 % (8 %)	34 % (15 %)
Framingham	34 % (15 %)	38 % (18 %)
ASCVD	25 % (14 %)	33 % (13 %)

Mean (SD), unless otherwise indicated. *Missing imaging parameters* (n) baseline/follow-up: TBV 9/11; WMH volume 9/7; ASL-CBF GM non-crushed 9/9; ASL-CBF GM crushed 9/9; ASL sCoV non-crushed 9/9; ASL sCoV crushed 9/9. *Missing cardiovascular risk scores* (n) baseline/follow-up: SCORE O.P. 1/19; Framingham 1/19, ASCVD 65/86.

### 3.1 Main analyses

Results of the cross-sectional linear regression analysis on baseline and follow-up scans are listed in Table 2, Table 3 respectively and scatterplots are visually presented in supplement Fig. 1. At baseline, higher SCORE O.P. atherosclerosis risk estimates were associated with worse perfusion in all models, both in the form of higher ASL-sCoV (model 3: non-crushed standardized-beta = 0.25, 95 %CI = 0.11 to 0.39, p = 0.0007; crushed standardized-beta = 0.22, 95 % confidence interval (CI) = 0.08 to 0.36, p = 0.002;) and lower ASL-CBF (model 3: non-crushed standardized-beta = -0.16, 95 %CI = -0.31 to −0.01, p = 0.03; crushed standardized-beta = -0.24, 95 %CI = -0.38 to −0.09, p = 0.001). These relations were attenuated at follow-up, although only slightly for ASL-sCoV (model 3: non-crushed standardized-beta = 0.15, 95 %CI = -0.04 to 0.34, p = 0.13; crushed standardized-beta = 0.17, 95 %CI = -0.01 to 0.35, p = 0.06), and more strongly for ASL-CBF (model 3; non-crushed standardized-beta = -0.07, 95 %CI = -0.27 to 0.13, p = 0.50; crushed standardized-beta = -0.15, 95 %CI = -0.34 to 0.05, p = 0.14). WMH and GM volume were not associated with the SCORE O.P. risk estimation in neither the baseline nor follow-up regression models.

**Table 2:** Results of standardized linear regression models on the association of **baseline** MRI parameters and baseline SCORE O.P. (n = 195).

	**Model 1, crude**			**Model 2, ‡**			**Model 3, §**		
**Variable**	**β -coefficient**	**95 %CI**	**p-value**	**β -coefficient**	**95 %CI**	**p-value**	**β -coefficient**	**95 %CI**	**p-value**
WMH volume *^,^†	0.09	−0.06 to 0.23	0.25	0.10	−0.04 to 0.26	0.18	0.13	−0.03 to 0.28	0.11
GM volume †	0.003	−0.05 to 0.05	0.09	−0.01	−0.16 to 0.14	0.89	−0.02	−0.17 to 0.13	0.79
ASL-CBF GM, non-crushed	−0.18	−0.32 to −0.04	**0.01**	−0.16	−0.31 to −0.02	**0.03**	−0.16	−0.31 to −0.01	**0.03**
ASL-CBF GM, crushed	−0.26	−0.40 to −0.13	**0.0002**	−0.24	−0.38 to −0.09	**0.001**	−0.24	−0.38 to −0.09	**0.001**
ASL spatial CoV, non-crushed *	0.25	0.12 to 0.39	**0.0002**	0.24	0.11 to 0.38	**0.0006**	0.25	0.11 to 0.39	**0.0007**
ASL spatial CoV, crushed *	0.23	0.10 to 0.36	**0.0006**	0.22	0.08 to 0.35	**0.002**	0.22	0.08 to 0.36	**0.002**

* These variables were introduced as a log scale. † Adjusted for TBV. ‡ Model 2 was adjusted for history of CVD and/or stroke. § Model 3 was adjusted for history of CVD and stroke, ethnicity, BMI, LDL, diastolic blood pressure. *Abbreviations*: TBV; total brain volume, WMH; white matter Hyperintensities, GM; gray matter, CBF; cerebral blood flow, ASL; arterial spin labeling, CoV; coefficient of variation.

**Table 3:** Results of standardized linear regression models on the association of **follow-up** MRI parameters and follow-up SCORE O.P. (n = 135).

	**Model 1, crude**			**Model 2, ‡**			**Model 3, §**		
**Variable**	**β -coefficient**	**95 %CI**	**p-value**	**β -coefficient**	**95 %CI**	**p-value**	**β -coefficient**	**95 %CI**	**p-value**
WMH volume *^,^†	0.10	−0.08 to 0.28	0.29	0.08	−0.10 to 0.27	0.38	0.09	−0.10 to0.28	0.35
GM volume †	0.004	−0.06 to 0.06	0.89	−0.005	−0.07 to 0.06	0.86	0.009	−0.08 to 0.05	0.71
ASL-CBF GM, non-crushed	−0.04	−0.22 to 0.14	0.64	−0.06	−0.26 to 0.13	0.52	−0.07	−0.27 to 0.13	0.50
ASL-CBF GM, crushed	−0.14	−0.33 to 0.04	0.12	−0.14	−0.34 to 0.05	0.15	−0.15	−0.34 to 0.05	0.14
ASL spatial CoV, non-crushed *	0.17	−0.002 to 0.35	0.06	0.14	−0.05 to 0.32	0.15	0.15	−0.04 to 0.34	0.13
ASL spatial CoV, crushed *	0.20	0.03 to 0.36	**0.02**	0.17	−0.01 to 0.34	0.07	0.17	−0.01 to 0.35	0.06

* These variables were introduced as a log scale. † Adjusted for TBV. ‡ Model 2 was adjusted for history of CVD and/or stroke. § Model 3 was adjusted for history of CVD and stroke, ethnicity, BMI, LDL, diastolic blood pressure. *Abbreviations*: TBV; total brain volume, WMH; white matter Hyperintensities, GM; gray matter, CBF; cerebral blood flow, ASL; arterial spin labeling, CoV; coefficient of variation.

### 3.2 Secondary analyses

Sensitivity analyses excluding all participants with a history of CVD and/or stroke and in a model with imputed values for missing SCORE O.P. risk estimates did not change the results of our main analyses (supplement table 1 and 2). An additional analysis excluding MRI imaging outliers showed a largely similar association to our primary analyses, except for a slightly stronger association for the SCORE O.P. with non-crushed ASL-sCoV on follow-up (supplement table 3). Analysis of relationships between baseline SCORE O.P. and baseline MRI parameters only in participants that had attended both the baseline and follow-up MRI yielded similar results as our main analysis, apart from a smaller, non-significant effect size for non-crushed ASL-CBF within this group (supplement table 4). Analysis of change in MRI parameters from baseline to follow-up in these participants did not suggest a relation with baseline SCORE O.P. (supplement table 5). The association between baseline atherosclerotic risk and follow-up MRI parameters was similar to our cross-sectional outcomes, with effect sizes between effect sizes of baseline and follow-up associations (supplement table 6). When excluding age from the SCORE O.P. risk equation, the mean SCORE O.P. was the same at baseline and follow-up (10 % ±4 risk of 10-year CVD). The association of the SCORE O.P. risk score with the examined MRI parameters was unaffected when age was not included in the calculation of the risk score (supplement table 7).

The final sensitivity analysis comparing results using the SCORE O.P. to those using two other commonly used scores to measure atherosclerosis risk (Framingham and ASCVD) showed similar associations for the SCORE O.P. and the Framingham risk estimations (supplement table 8). There was no association for ASCVD with ASL-sCoV (crushed) on baseline and follow-up, although the power of this analysis was limited due to a high number of missing values (33 % missing on baseline, 64 % on follow-up) as the ASCVD risk scores require risk-factor data from two consecutive visits.

## 4 Discussion

The main findings of this study are twofold. First, our results show that lower GM ASL-CBF and higher ASL-sCoV were cross-sectionally associated with a higher estimated 10-year risk of cardiovascular disease risk in community-dwelling older people, while WMH volume was not significantly associated with this risk. Second, sensitivity analyses on the association between longitudinal changes in investigated MRI parameters did not show a clear relation of change in these parameters over 2.8 years of follow-up with atherosclerotic risk scores. This suggests that ASL-derived parameters, in particular ASL-sCoV, may be more sensitive markers of cerebrovascular disease than WMH volume but may be less sensitive to its longitudinal course.

Our findings are in line with previous research demonstrating the relation between cardiovascular risk and ASL-CBF (Anand et al., 2019, Pase et al., 2012, Suri et al., 2019) and add evidence of an often even stronger correlation of these risk factors with the new ASL-sCoV. ASL-sCoV may be a more sensitive parameter for cerebrovascular pathology than ASL-CBF, as it reflects the efficiency with which labeled blood can be delivered from the neck to the imaging voxel and therefore might correspond better to cerebrovascular resistance while ASL-CBF better reflects tissue perfusion (Mutsaerts et al., 2017, Mutsaerts et al., 2020, Ibaraki et al., 2019). Additionally, ASL-sCoV is calculated by dividing the standard deviation of ASL-CBF by its mean across the whole brain. Therefore, ASL-sCoV has reduced sensitivity to global perfusion values and lower sensitivity to variability in labeling efficiency, which may have enhanced its statistical power to show differences between individuals compared with ASL-CBF. This might have been slightly aggravated in the present study because of the missing M0 scans, as M0 scans normally reduce global CBF variability due to inter-individual variability of M0 and regional M0 changes caused by imperfect repositioning. Conversely, variability of labeling efficiency between individuals potentially leads to higher intra-individual variability of ASL-CBF when compared to ASL-sCoV.

In this study, ASL-sCoV from crushed and non-crushed ASL had a similar ability to detect changes in atherosclerotic risk scores, implying that mild vascular crushing — i.e. removing blood flow with a velocity higher than 5 cm/s to filter out the ASL-CBF in the large vessels — does not have a large effect on capturing the distribution of labeled blood. Nevertheless, our non-crushed ASL-CBF values did have lower correlations with atherosclerotic risk scores than the crushed ASL-CBF values, suggesting that the microvascular ASL signal in the crushed images is an important component of the correlation between ASL-CBF estimates and atherosclerotic risk scores.

Follow-up ASL-CBF and ASL-sCoV were less correlated with atherosclerotic risk in this study, which could be explained because some risk factors for the SCORE O.P. that were measured at baseline (e.g. SBP, cholesterol, smoking) may have been under better control or treatment at follow-up as a result of created awareness, resulting in relatively lower SCORE O.P. scores, while the effects of chronic atherosclerosis on ASL-CBF and ASL-sCoV remain, which potentially slightly attenuated relations in follow-up analyses. The relation between baseline SCORE O.P. scores and follow-up MRI parameters was comparable to the relation between follow-up SCORE O.P. scores and follow-up MRI parameters, suggesting that this decrease in effect size was not evidently driven by change in MRI parameters from baseline to follow-up.

Similar to previous research, we did not observe an association between longitudinal change in global ASL-CBF with atherosclerotic risk scores in this study (Glodzik et al., 2011). A possible explanation could be that global ASL-CBF is not sensitive enough to detect these changes over time, as significant associations with cardiovascular disease have previously only been demonstrated for differences in regional, not global, ASL-CBF (Suri et al., 2019, Bangen et al., 2014). To allow for comparison between CBF and ASL-sCoV, we did not investigate regional ASL-CBF in this study, because ASL-sCoV can only be obtained for large areas including both proximal and distal vasculature, it being a measure of the spatial distribution of the ASL-signal (Beason-Held et al., 2012). Another potential explanation for the absence of a longitudinal association between global ASL-CBF and baseline atherosclerotic risk scores may be that the relatively short time interval of our longitudinal analyses (with a mean of 2.8 years) was insufficient to study the longitudinal relation of ASL-CBF and ASL-sCoV with atherosclerotic risk. Alternatively, atherosclerotic risk scores may be better differentiators of atherosclerosis between individuals at a single time-point, than of change in atherosclerosis within older individuals over a relatively short time span. Combined with the high physiological variability in ASL-CBF (Clement et al., 2018), examining relations with ASL-CBF over time in this study is challenging. Therefore, it may be interesting to investigate the added value of ASL-sCoV assessments over time in future (pooled) studies with a large scale of different time points.

WMH volume and atherosclerosis share several risk factors (e.g. hypertension, diabetes mellitus, dyslipidemia), however, WMH volume was not associated with the estimated 10-year cardiovascular risk in our study. A possible explanation could be that, as WMHs occur as a later consequence of atherosclerosis, concomitant increased vascular risk does not correlate very well with WMH volume because the process of atherosclerosis resulting in WMHs is still ongoing (de Leeuw et al., 1999, White et al., 2011, Verhaaren et al., 1979). Also, however the association of WMH volume with mid-life cardiovascular risk factors has been established before, the findings of this and previous studies suggest that these risk factors are potentially less discriminative in late-life (Gattringer et al., 2012, Dickie et al., 1936). Finally, the relation between WMH and atherosclerotic risk scores was not significant in our study, but standardized effect sizes were consistently positive and it is conceivable that significant associations for WMH volume would have been observed with a substantially larger sample size. However, given that we did find significant associations for the current sample size with ASL-derived parameters, our findings suggest that these parameters may be much more sensitive markers of atherosclerotic risks than WMH volume.

The strengths of this study are the repeated ASL scans in a relatively large sample of cognitively intact older participants and the precise and repeated evaluation of their vascular risk profiles. Our study has several limitations. First, although comparisons of MRI parameters and cardiovascular risk at baseline and follow-up demonstrated similar effect sizes, there was limited power within our analyses on follow-up scans. Second, no explicit restrictions were enforced on participant conditions that could influence ASL-CBF (e.g. medication and caffeine use). This could potentially contribute to the high physiological variability of ASL-CBF measurements in individuals, however, this should not introduce a systemic bias on the group level (Clement et al., 2018, Joris et al., 2018). Furthermore, a substantial number of our participants had a history of CVD or stroke at baseline. Atherosclerotic risk scores are not validated for these participants, however, we corrected for these covariates in our adjusted models and our sensitivity analyses excluding participants with a history of CVD or stroke did not change our findings. In addition, up to 40 % of our participants were using antihypertensive drugs. The SCORE O.P. risk incorporates systolic blood pressure without accounting for antihypertensive treatment, thereby potentially underestimating the true atherosclerotic risk for participants using antihypertensive drugs. The Framingham risk score does include antihypertensive drug usage, which is reflected in a higher 10-year risk in our population, however, this did not affect the association of MRI parameters and risk scores in our analyses. Another limitation of this study is missing information on intra- or extracranial stenosis, which could potentially influence ATT. However, a relatively healthy population of community-dwelling older people who were not selected based on previous cardiovascular conditions was studied and the prevalence of significant intra- or extracranial stenosis is low in such a general population (Prati et al., 2006). Therefore, we expect that the potential presence of stenosis has a limited impact on our findings. Furthermore, because M0 acquisition was not available, we used a mean deriver M0-value obtained previously from the same sequence to calibrate our scans. While this approach is not optimal, it is likely more stable than using the control images with background suppression. A limitation of this approach is that the B1-field inhomogeneities are not compensated for when using a single global value of blood T1, however, this should not affect our study results that work with whole-brain ASL-CBF values. Lastly, by assessing the relation between MRI parameters and atherosclerotic risk scores, we only used a proxy of cerebrovascular atherosclerosis. Magnetic resonance vessel-wall imaging could probably provide a more precise estimation of the extent of atherosclerosis than atherosclerotic risk scores, but we could not investigate this with our current data.

Our findings demonstrate that ASL-CBF and ASL-sCoV correlate better with atherosclerotic risk scores in older adults than the more conventional small vessel disease marker of WMH volume. ASL-sCoV appeared to be even stronger correlated with atherosclerotic risk score than the more commonly used ASL-CBF, though this difference was modest. Recent guidelines on cardiovascular prevention stress the need for additional risk stratification markers beyond risk scores in cases of clinical uncertainty and patient selection (Lloyd-Jones et al., 2017). Our data reaffirm that non-invasive imaging with MRI is highly informative and could provide additional information about cerebrovascular damage, potentially becoming a well-defined stratification marker for participants in whom early prevention of atherosclerosis and cardiovascular disease might still be attainable (Anand et al., 2019). Future research should re-affirm these findings by comparing ASL MRI parameters with definite markers of atherosclerosis, such as MRI vessel wall thickness, neuropathology, or cerebrovascular events. Additionally, it would be interesting to investigate if regional CBF changes could potentially be even more strongly correlated with atherosclerotic risk. Longitudinal studies could look at the association between repeated ASL measurements and atherosclerosis, and potentially even look into the predictive value of these MRI parameters for incident stroke and cardiovascular disease. Lastly, more advanced ASL imaging methods, such as a multi-delay acquisition protocol, could help to address the issue with significant variations in ATT across subjects and over time, allowing for more accurate estimations of ASL-CBF and -sCoV (Wang et al., 2013, Li et al., 2015).

## 5 Disclosures

The authors have no conflicts of interest to disclose. The corresponding author (MH) had full access to all the data in the study and takes responsibility for its integrity and the data analysis.

## 6 Funding acknowledgments

The author(s) disclosed receipt of the following financial support for the research, authorship, and/or publication of this article: The preDIVA trial was supported by the Dutch Ministry of Health, Welfare and Sport (grant number 50-50110-98-020), the Dutch Innovation Fund of Collaborative Health Insurances (grant number 05-234), and the Netherlands Organization for Health Research and Development (grant number 62000015). The preDIVA MRI substudy was additionally funded by Internationale Stichting Alzheimer Onderzoek (grant number 10157). None of the funding sources had any involvement in the design of the study or in the collection, analysis, and interpretation of the data.

## 7 Author contribution statement

Conception and design of the study: HJJM, ER, JWvD. Acquisition and analysis of data: MH, HJMM, JP, JWvD. Drafting a significant portion of the manuscript or figures: MH, HJMM, JP, ER, JWvD.

All authors meet the criteria for authorship stated in the Uniform Requirements for Manuscripts Submitted to Biomedical Journals and all authors have read and approved the final version submitted.

## CRediT authorship contribution statement

**Melanie Hafdi:** Writing – original draft, Methodology, Formal analysis, Visualization. **Henk JMM Mutsaerts:** Writing – review & editing, Conceptualization, Methodology, Data curation. **Jan Petr:** Writing – review & editing, Conceptualization, Methodology, Data curation. **Edo Richard:** Writing – review & editing, Conceptualization, Methodology. **Jan Willem van Dalen:** Writing – review & editing, Conceptualization, Methodology, Data curation, Supervision.

## Declaration of Competing Interest

The authors declare that they have no known competing financial interests or personal relationships that could have appeared to influence the work reported in this paper.
