# Supplementary material for: Atherosclerotic risk is associated with cerebral perfusion – A cross-sectional study using arterial spin labeling MRI
Source: Neuroimage Clin. 2022 Aug 4;36:103142. doi: 10.1016/j.nicl.2022.103142 (PMC9400119; doi:10.1016/j.nicl.2022.103142)
Supplement: Supplementary data 1 [file mmc1.docx]

**Supplementary materials**

Atherosclerotic risk is associated with cerebral perfusion

**Supplement table 1**. Sensitivity analysis: Results of standardized linear regression models on the association of baseline and follow-up MRI parameters and SCORE O.P. in participants without a history of cardiovascular disease (CVD) and/or stroke

|  | **Without history of CVD** | | | | **Without history of CVD and stroke** | | | |
| --- | --- | --- | --- | --- | --- | --- | --- | --- |
|  | Baseline – crude  (n=149) | | Follow-up – crude  (n=146) |  | Baseline – crude  (n=139) | | Follow-up – crude  (n=135) |  |
| **Variable** | **β (95%CI)** | **P-value** | **β (95%CI)** | **p-value** | **β (95%CI)** | **p-value** | **β (95%CI)** | **p-value** |
| WMH volume *^,^† | 0.05 (-0.11-0.21) | 0.54 | 0.08 (-0.13-0.29) | 0.46 | 0.07 (-010-0.24) | 0.41 | 0.10 (-0.11-0.32) | 0.35 |
| GM volume † | 0.01 (-0.04-0.06) | 0.77 | 0.01 (-0.05-0.07) | 0.79 | 0.01 (-0.05-0.06) | 0.84 | 0.01 (-0.06-0.07) | 0.86 |
| ASL-CBF GM, non-crushed | -0.16 (-0.32- -0.01) | **0.04** | -0.02 (-0.24-0.20) | 0.87 | -0.17 (-0.34- -0.01) | **0.04** | -0.02 (-0.25-0.21) | 0.85 |
| ASL-CBF GM, crushed | -0.26 (-0.41- -0.10) | **0.001** | -0.07 (-0.29-0.14) | 0.50 | -0.26 (-0.42- -0.10) | **0.002** | -0.07 (-0.29-0.16) | 0.57 |
| ASL spatial CoV, non-crushed * | 0.29 (0.14-0.43) | **0.0002** | 0.08 (-0.12-0.29) | 0.43 | 0.31 (0.16-0.46) | **> 0.0001** | 0.10 (-0.12-0.31) | 0.39 |
| ASL spatial CoV, crushed * | 0.26 (0.12-041) | **0.0005** | 0.11 (-0.09-0.32) | 0.28 | 0.28 (0.14-0.43) | **0.0003** | 0.13 (-0.09-0.34) | 0.24 |

Baseline analyses are the relation between baseline SCORE O.P. with contemporary baseline MRI, follow-up analyses are the relation between follow-up SCORE O.P. with contemporary follow-up MRI. * These variables were introduced as a log scale. † Adjusted for TBV. *Abbreviations:* TBV; total brain volume. CBF; cerebral blood flow, GM; gray matter, ASL; arterial spin labeling, CoV; coefficient of variation.

|  | **Baseline** | | **Follow-up** | |
| --- | --- | --- | --- | --- |
|  | **Reference** | **Imputed crude** | **Reference** | **Imputed crude** |
| **Variable** | **β (95%CI)** | **β (95%CI)** | **β (95%CI)** | **β (95%CI)** |
| WMH volume *^,^† | 0.09 (-0.06-0.23) | 0.09 (-0.05-0.23) | 0.10 (-0.08-0.28) | 0.11 (-0.03-0.25) |
| GM volume † | 0.003 (-0.05-0.05) | 0.002 (-0.05-0.05) | 0.004 (-0.06-0.06) | 0.004 (-0.06-0.07) |
| ASL-CBF GM, non-crushed | -0.18 (-0.32- -0.04) | -0.18 (-0.32- -0.04) | -0.04 (-0.22-0.14) | 0.03 (-0.12-0.17) |
| ASL-CBF GM, crushed | -0.26 (-0.40- -0.13) | -0.27 (-0.41- -0.13) | -0.14 (-0.33-0.04) | -0.07 (-0.21-0.08) |
| ASL spatial CoV, non-crushed * | 0.25 (0.12-0.39) | 0.25 (0.11-0.40) | 0.17 (-0.002-0.35) | 0.13 (-0.03-0.29) |
| ASL spatial CoV, crushed * | 0.23 (0.10-0.36) | 0.23 (0.09-0.37) | 0.20 (0.03-0.36) | 0.15 (-0.01-0.31) |

**Supplement table 2**. Sensitivity analysis: Results of imputed standardized linear regression models on the association of baseline and follow-up MRI parameters with SCORE O.P.

Reference outcomes are results from model 1 (crude analysis) of the main analysis. Baseline analyses are the relation between baseline SCORE O.P. with contemporary baseline MRI, follow-up analyses are the relation between follow-up SCORE O.P. with contemporary follow-up MRI. * These variables were introduced as a log scale. † Adjusted for TBV.
*Abbreviations:* TBV; total brain volume, WMH; white matter hyperintensity, CBF; cerebral blood flow, GM; gray matter, ASL; arterial spin labeling, CoV; coefficient of variation.

**Supplement table 3**. Sensitivity analysis: Results of standardized linear regression models on the association of baseline and follow-up MRI parameters with baseline and follow-up SCORE O.P. respectively without MRI outliers (defined as Q1-Q3 +/- 1.5*IQR

|  | **Baseline** | | | | **Follow-up** | | | |
| --- | --- | --- | --- | --- | --- | --- | --- | --- |
|  | Reference | | Without outliers |  | Reference | | Without outliers |  |
| **Variable** | **β (95%CI)** | **P-value** | **β (95%CI)** | **p-value** | **β (95%CI)** | **p-value** | **β (95%CI)** | **p-value** |
| WMH volume *^,^† | 0.09 (-0.06-0.23) | 0.25 | 0.06 (-0.08-0.21) | 0.39 | 0.10 (-0.08-0.28) | 0.29 | 0.08 (-0.10-0.002) | 0.40 |
| GM volume † | 0.003 (-0.05-0.05) | 0.90 | 0.004 (-0.05-0.06) | 0.88 | 0.004 (-0.06-0.06) | 0.89 | 0.005 (-0.06-0.8) | 0.88 |
| ASL-CBF GM, non-crushed | -0.18 (-0.32- -0.04) | **0.01** | -0.16 (-0.30- -0.01) | **0.04** | -0.04 (-0.22-0.14) | 0.64 | 0.02 (-0.18-0.22) |  |
| ASL-CBF GM, crushed | -0.26 (-0.40- -0.13) | **0.0002** | -0.24 (-0.38- -0.10) | **0.001** | -0.14 (-0.33-0.04) | 0.12 | -0.15 (-0.34-0.04) | 0.13 |
| ASL spatial CoV, non-crushed * | 0.25 (0.12-0.39) | **0.0002** | 0.27 (0.13-0.41) | **0.0003** | 0.17 (-0.002-0.35) | 0.06 | 0.21 (0.01-0.40) | **0.04** |
| ASL spatial CoV, crushed * | 0.23 (0.10-0.36) | **0.0006** | 0.24 (0.10-0.39) | **0.0009** | 0.20 (0.03-0.36) | **0.02** | 0.21 (0.02-0.40) | **0.04** |

Reference outcomes are results from model 1 (crude analysis) of the main analysis. Baseline analyses are the relation between baseline SCORE O.P. with contemporary baseline MRI, follow-up analyses are the relation between follow-up SCORE O.P. with contemporary follow-up MRI. * These variables were introduced as a log scale. † Adjusted for TBV.
*Abbreviations*: TBV; total brain volume, WMH; white matter Hyperintensities, GM; gray matter, CBF; cerebral blood flow, ASL; arterial spin labeling, CoV; coefficient of variation.

**Supplement table 4.** Sensitivity analysis: Results of standardized linear regression model on the association of baseline MRI parameters with SCORE O.P. for participants on **baseline** that have also attended the follow-up scan (n=135)

|  | **Model 1, crude** | | | **Model 2, ‡** | | **Model 3,** **§** | |
| --- | --- | --- | --- | --- | --- | --- | --- |
| **Variable** | **β (95%CI)** | | **p-value** | **β (95%CI)** | **p-value** | **β (95%CI)** | **p-value** |
| WMH volume *^,^† | | 0.11 (-0.06-0.29) | 0.19 | 0.12 (-0.07-0.30) | 0.22 | 0.15 (-0.04-0.34) | 0.13 |
| GM volume † | | 0.01 (-0.04-0.07) | 0.63 | 0.001 (-0.06-0.06) | 0.98 | -0.005 (-0.07-0.06) | 0.88 |
| ASL-CBF GM, non-crushed | | -0.13 (-0.29-0.04) | 0.14 | -0.07 (-0.25-0.11) | 0.43 | -0.06 (-0.24-0.12) | 0.52 |
| ASL-CBF GM, crushed | | -0.23 (-0.39- -0.06) | **0.008** | -0.16 (-0.34-0.01) | 0.07 | -0.15 (-0.33-0.02) | 0.09 |
| ASL spatial CoV, non-crushed * | | 0.22 (0.06-0.38) | **0.007** | 0.18 (0.01-0.35) | **0.04** | 0.18 (0.004-0.35) | **0.05** |
| ASL spatial CoV, crushed * | | 0.20 (0.05-0.36) | **0.01** | 0.16 (-0.01-0.33) | 0.07 | 0.15 (-0.02-0.32) | 0.08 |

* These variables were introduced as a log scale. † Adjusted for TBV‡ Model 2 was adjusted for history of CVD and/or stroke. § Model 3 was adjusted for history of CVD and stroke, ethnicity, BMI, LDL, diastolic blood pressure. *Abbreviations*: TBV; total brain volume, WMH; white matter Hyperintensities, GM; gray matter, CBF; cerebral blood flow, ASL; arterial spin labeling, CoV; coefficient of variation.

**Supplement table 5.** Results of standardized linear regression model on the association of absolute change in MRI parameter between baseline and follow-up and baseline SCORE O.P.

|  | **Model 1, crude** | |
| --- | --- | --- |
| **Variable** | **β (95%CI)** | **p-value** |
| WMH volume * | 0.13 (-0.04-0.30) | 0.13 |
| GM volume * | -0.004 (-0.11-0.10) | 0.95 |
| ASL-CBF GM, non-crushed | 0.05 (-0.12-0.23) | 0.57 |
| ASL-CBF GM, crushed | 0.07 (-0.11-0.24) | 0.46 |
| ASL spatial CoV, non-crushed | 0.03 (-0.14-0.20) | 0.74 |
| ASL spatial CoV, crushed | 0.05 (-0.11-0.20) | 0.53 |

* Adjusted for difference in TBV. *Abbreviations*: TBV; total brain volume, WMH; white matter Hyperintensities, GM; gray matter, CBF; cerebral blood flow, ASL; arterial spin labeling, CoV; coefficient of variation.

**Supplement table 6**. Results of standardized linear regression model on the association of the baseline SCORE O.P. with follow-up MRI parameters.

|  | **Reference** | | **Baseline SCORE O.P,  FU MRI Parameter** |
| --- | --- | --- | --- |
| **Variable** | **BASELINE –**  **β (95%CI)** | **FOLLOW-UP –**  **β (95%CI)** | **β (95%CI)** |
| WMH volume *^,^† | 0.09 (-0.06-0.23) | 0.63 (-0.51-1.77) | 0.11 (-0.06-0.28) |
| GM volume † | 0.003 (-0.05-0.05) | 1.70 (-21.95-25.36) | 0.01 (-0.05-0.07) |
| ASL-CBF GM, non-crushed | -0.18 (-0.32- -0.04) | -7.53 (-39.18-24.13) | -0.04 (-0.21-0.14) |
| ASL-CBF GM, crushed | -0.26 (-0.40- -0.13) | -24.73 (-55.90-6.43) | -0.13 (-0.31-0.04) |
| ASL spatial CoV, non-crushed * | 0.25 (0.12-0.39) | 0.33 (-0.003-0.67) | 0.18 (0.01-0.35) |
| ASL spatial CoV, crushed * | 0.23 (0.10-0.36) | 0.38 (0.03-0.71) | 0.21 (0.05-0.37) |

Reference outcomes are results from model 1 (crude analysis) of the main analysis. * These variables were introduced as a log scale. † Adjusted for TBV.
*Abbreviations*: TBV; total brain volume, WMH; white matter Hyperintensities, GM; gray matter, CBF; cerebral blood flow, ASL; arterial spin labeling, CoV; coefficient of variation.

**Supplement table 7**. Sensitivity analysis: Results of linear regression models on the association of baseline and follow-up MRI parameters with baseline and follow-up SCORE O.P. respectively excluding age from the SCORE O.P. risk equation

|  |  | **Reference** | | **Without age** | |
| --- | --- | --- | --- | --- | --- |
| **Baseline** | **Variable** | **β (95%CI)** | **p-value** | **β (95%CI)** | **p-value** |
|  | WMH volume *^,^† | 0.09 (-0.06-0.23) | 0.25 | -0.01 (-0.17-0.14) | 0.84 |
|  | GM volume † | 0.003 (-0.05-0.05) | 0.91 | -0.01 (-0.06-0.04) | 0.63 |
|  | ASL-CBF GM, non-crushed | -0.18 (-0.32- -0.04) | **0.01** | -0.12 (-0.26-0.02) | 0.09 |
|  | ASL-CBF GM, crushed | -0.26 (-0.40- -0.13) | **0.0002** | -0.23 (-0.37- -0.09) | **0.001** |
|  | ASL spatial CoV, non-crushed * | 0.25 (0.12-0.39) | **0.0002** | 0.20 (0.07-0.34) | **0.004** |
|  | ASL spatial CoV, crushed * | 0.23 (0.10-0.36) | **0.0006** | 0.21 (0.08-0.34) | **0.002** |
| **Follow-up** | WMH volume *^,^† | 0.10 (-0.08-0.28) | 0.39 | -0.04 (-0.21-0.15) | 0.69 |
|  | GM volume † | 0.004 (-0.06-0.06) | 0.89 | -0.02 (-0.08-0.04) | 0.57 |
|  | ASL-CBF GM, non-crushed | -0.04 (-0.22-0.14) | 0.64 | 0.18 (0.01-0.36) | **0.04** |
|  | ASL-CBF GM, crushed | -0.14 (-0.33-0.04) | 0.12 | 0.07 (-0.11-0.24) | 0.47 |
|  | ASL spatial CoV, non-crushed * | 0.17 (-0.002-0.35) | 0.06 | 0.10 (-0.07-0.27) | 0.25 |
|  | ASL spatial CoV, crushed * | 0.20 (0.03-0.36) | **0.02** | 0.12 (-0.05-0.28) | 0.17 |

Reference outcomes are results from model 1 (crude analysis) of the main analysis. Baseline analyses are the relation between baseline SCORE O.P. with contemporary baseline MRI, follow-up analyses are the relation between follow-up SCORE O.P. with contemporary follow-up MRI. * These variables were introduced as a log scale. † Adjusted for TBV.
*Abbreviations*: TBV; total brain volume, WMH; white matter Hyperintensities, GM; gray matter, CBF; cerebral blood flow, ASL; arterial spin labeling, CoV; coefficient of variation.

**Supplement table 8**. Results of linear regression models for the associations of baseline and follow-up MRI parameters with 3 different CVD risk models

|  |  | **SCORE O.P.** | | **Framingham** | | **ASCVD** | |
| --- | --- | --- | --- | --- | --- | --- | --- |
| **Baseline** | **Variable** | **β (95%CI)** | **p-value** | **β (95%CI)** | **p-value** | **β (95%CI)** | **p-value** |
|  | WMH volume *^,^† | 0.09 (-0.06-0.23) | 0.25 | 0.04 (-0.11-0.19) | 0.59 | 0.12 (-0.05-0.29) | 0.16 |
|  | GM volume † | 0.003 (-0.05-0.05) | 0.91 | -0.01 (-0.06-0.04) | 0.8 | -0.02 (-0.08-0.04) | 0.58 |
|  | ASL-CBF GM, non-crushed | -0.18 (-0.32- -0.04) | **0.01** | -0.14 (-0.28- -0.003) | **< 0.05** | -0.24 (0.43- -0.06) | **0.01** |
|  | ASL-CBF GM, crushed | -0.26 (-0.40- -0.13) | **0.0002** | -0.23 (-0.37- -0.10) | **0.001** | -0.20 (-0.38- -0.02) | **0.03** |
|  | ASL spatial CoV, non-crushed * | 0.25 (0.12-0.39) | **0.0002** | 0.24 (0.11-0.37) | **0.0005** | 0.16 (0.002-0.32) | **< 0.05** |
|  | ASL spatial CoV, crushed * | 0.23 (0.10-0.36) | **0.0006** | 0.24 (0.11-0.37) | **0.0004** | 0.11 (-0.05-0.27) | 0.19 |
| **Follow-up** | WMH volume *^,^† | 0.10 (-0.08-0.28) | 0.39 | 0.04 (-0.15-0.23) | 0.67 | 0.22 (-0.07-0.50) | 0.15 |
|  | GM volume † | 0.004 (-0.06-0.06) | 0.89 | -0.01 (-0.07-0.05) | 0.74 | -0.07 (-0.16-0.02) | 0.13 |
|  | ASL-CBF GM, non-crushed | -0.04 (-0.22-0.14) | 0.64 | 0.12 (-0.06-0.30) | 0.19 | 0.16 (-0.12-0.45) | 0.27 |
|  | ASL-CBF GM, crushed | -0.14 (-0.33-0.04) | 0.12 | -0.01 (-0.20-0.17) | 0.87 | 0.03 (-0.25-0.32) | 0.81 |
|  | ASL spatial CoV, non-crushed * | 0.17 (-0.002-0.35) | 0.06 | 0.13 (-0.04-0.30) | 0.14 | 0.01 (-0.22-0.24) | 0.93 |
|  | ASL spatial CoV, crushed * | 0.20 (0.03-0.36) | **0.02** | 0.16 (-0.002-0.33) | 0.06 | -0.02 (-0.26-0.21) | 0.84 |

Baseline analyses are the relation between baseline SCORE O.P. with contemporary baseline MRI, follow-up analyses are the relation between follow-up SCORE O.P. with contemporary follow-up MRI. * These variables were introduced as a log scale. † Adjusted for TBV. SCORE O.P.: 10 year CVD and CHD risk, Framingham 10 year CVD and CHD risk, ASCVD 10 year CVD risk. *Abbreviations*: TBV; total brain volume, WMH; white matter Hyperintensities, GM; gray matter, CBF; cerebral blood flow, ASL; arterial spin labeling, CoV; coefficient of variation.

**
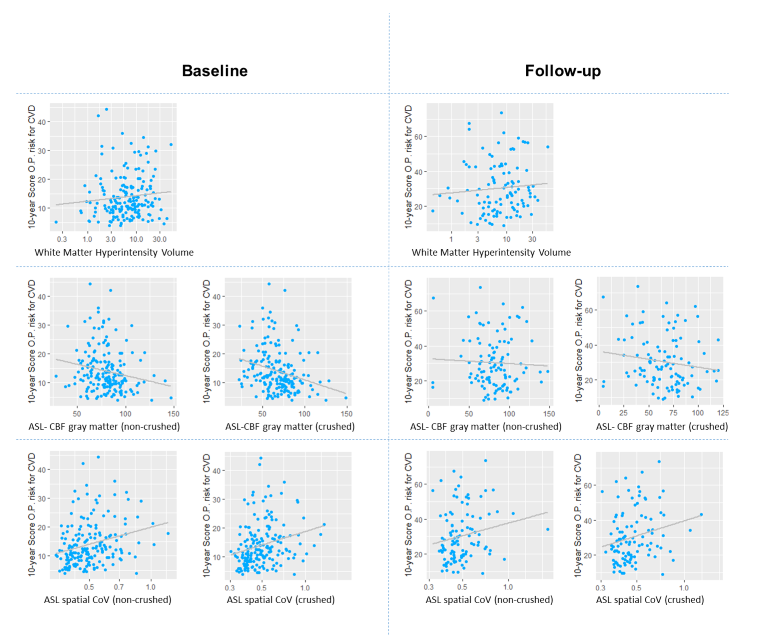
**

**Supplement figure 1.** Scatterplots MRI parameters and SCORE O.P.
Baseline figures depict baseline MRI values and baseline SCORE O.P. values, follow-up analyses depict follow-up MRI values and follow-up SCORE O.P. values.
CBF; cerebral blood flow in mL/100mg/min, ASL spatial CoV; arterial spin labeling spatial coefficient of variation as fraction.
